# Domestic Sautéing with EVOO: Change in the Phenolic Profile

**DOI:** 10.3390/antiox9010077

**Published:** 2020-01-16

**Authors:** Julián Lozano-Castellón, Anna Vallverdú-Queralt, José Fernando Rinaldi de Alvarenga, Montserrat Illán, Xavier Torrado-Prat, Rosa Maria Lamuela-Raventós

**Affiliations:** 1 Nutrition, Food Science and Gastronomy Department, XaRTA, Institute of Nutrition and Food Safety (INSA-UB), School of Pharmacy and Food Sciences, University of Barcelona, 08028 Barcelona, Spain; julian.lozano@ub.edu (J.L.-C.); avallverdu@ub.edu (A.V.-Q.); millan@ub.edu (M.I.); xaviertorrado@ub.edu (X.T.-P.); 2 CIBER Physiopathology of Obesity and Nutrition (CIBEROBN), Institute of Health Carlos III, 28029 Madrid, Spain; 3 Department of Food Science and Experimental Nutrition, School of Pharmaceutical Sciences, Food Research Center (FoRC), University of São Paulo, 05508-060 São Paulo, Brazil; zehfernando@gmail.com

**Keywords:** home-cooking, extra virgin olive oil, UPLC-ESI-QqQ-MS/MS, healthy cooking, Mediterranean diet

## Abstract

(1) Background: The health benefits of extra-virgin olive oil (EVOO), a key component of the Mediterranean diet, are attributed to its polyphenol profile. EVOO is often consumed cooked, and this process may degrade and transform polyphenols. (2) Methods: In this work, we determined how temperature, time, and the interaction between them affects the EVOO polyphenolic profile during a domestic pan-frying process, simulating the cooking conditions of a home kitchen, without the control of light or oxygen. Applying a 2^2^ full factorial design experiment, “Hojiblanca” EVOO was processed at two temperatures (120 °C and 170 °C) either for a short time or a long time, mimicking a domestic process, and polyphenol content was analyzed by UPLC-ESI-QqQ-MS/MS. (3) Results: Temperature degraded the polyphenols of EVOO during the sauté cooking process, whereas time had an effect on some individual phenols, such as hydroxytyrosol, but not on the total phenol content. The polyphenol content decreased by 40% at 120 °C and 75% at 170 °C compared to raw EVOO. (4) Conclusions: Cooked EVOO still meets the parameters of the EU’s health claim.

## 1. Introduction

Extra virgin olive oil (EVOO), the main source of fat in a Mediterranean diet, displays a singular fatty acid composition with a higher content of phenolic compounds and other antioxidants than other edible oils. Its health benefits are mainly attributed to these minor components, above all to simple phenols and polyphenols (both referred to henceforth as polyphenols) [1]. Its consumption has shown to play a protective role against a wide range of diseases [1,2], such as cancer [3], cardiovascular diseases [4], neurodegeneration [5], and diabetes [6]. EVOO phenolic concentration can be improved by changing agronomic and technical factors, such as the simple minimization of bruising by a selection of the variety [7].

The problem is that the Mediterranean consumption of EVOO is not only carried out by using it as a final seasoning; EVOO is also used in Mediterranean cuisine for roasting, sautéing (pan-frying), stir-frying, and deep-frying. All of these culinary techniques are thermal processes that could diminish the minor components of EVOO, such as polyphenols, by substances leaching (especially of more polar compounds) into the medium or by the degradation and transformation of its polyphenol content [8,9]. In addition to the loss of antioxidants, pro-oxidants formation can occur, especially when cooking at high temperatures, notably as a consequence of the lipid oxidation [10,11]. Nevertheless, EVOO polyphenols have been shown to reduce the heat-induced formation of undesired compounds, such as the cancerogenic heterocyclic amines [12], and the formation of acrolein and hexanal [13]. Finally, the polyphenols can act as lipid-derived carbonyl scavengers [14].

Most of the studies on cooking-induced changes in the polyphenol composition of EVOO have been carried out in laboratory conditions [15,16], applying non-conventional Mediterranean cooking techniques, like microwaving [17], or exploring the addition of a phenolic extract rather than EVOO [18]. Their results may not match those produced in a domestic setting because of the differences in oxygen and light availability or because polyphenol degradation in EVOO may be influenced by its content of other minor compounds [19]. 

On the other hand, previous studies carried out under more true-to-life conditions have focused on comparing the polyphenol content between raw and cooked foods [20] and between foods prepared with different cooking techniques [9]. However, they were not focused on evaluating EVOO polyphenol degradation or how this is affected by cooking factors, like temperature or time. When cooking factors, such as time, were explored, oil was heated for a longer time than the real cooking time, i.e., for 25 or even 36 h [21,22]. Furthermore, some works explored the degradation of total polyphenols measured by the Folin-Ciocalteau method, which is not selective and measures all antioxidant compounds [23]. Consequently, more research is required to determine the extent to which the loss of polyphenols during cooking is counteracted by the beneficial effects of EVOO, or how the phenolic profiles are altered during domestic cooking.

In this context, the aim of the present study was to determine changes in the EVOO polyphenolic profile during a domestic sautéing process commonly used in the Mediterranean diet [24], using a 2^2^ full factorial design to assess the effect of time, temperature, and the interactions between these two factors, mimicking real conditions (without oxygen or light control). The polyphenolic profile was measured using ultra-high performance liquid chromatography coupled to a tandem mass spectrometer detector (UPLC-ESI-QqQ-MS/MS), providing information on how the polyphenolic profile changed and how individual polyphenols degraded at different rates. 

## 2. Materials and Methods 

### 2.1. Chemicals and Standards

Acetonitrile, methanol, formic acid, and acetic acid were purchased from AppliChem, Panreac Quimica SA (Barcelona, Spain). Hexane, *p*-coumaric acid, ferulic acid, luteolin, oleuropein, oleocanthal, and pinoresinol were purchased from Sigma-Aldrich (St. Louis, MO, USA). Hydroxytyrosol was acquired from Extrasynthese (Genay, France) and apigenin from Fluka (St. Louis, MO, USA). Ultrapure water was obtained using a Milli-Q purification system (Millipore, Bedford, MA, USA).

### 2.2. Samples

Polyphenol degradation was assessed in the common Spanish “Hojiblanca” variety of EVOO, which has a medium concentration of polyphenols [25]. It was provided by the Fundación Patrimonio Comunal Olivarero and was produced from olives milled in December 2016 in Spain.

### 2.3. Domestic Sauté Process

To simulate the home-cooking process of sauté, EVOO was heated in a pan (20 cm diameter, 0.8 mm thickness, stainless steel 18/10, Excalibur, Pujadas, Girona, Spain), and the influence of the cooking process on polyphenol degradation was monitored at two different temperatures: moderate (120 °C) and high (170 °C). In order to assess the influence of time, short and long cooking times were determined for each temperature, corresponding to the time needed to obtain “al dente” and well-cooked textures, respectively. For determining these times, 200 g of potatoes and 100 g of chicken (an average portion) were pan-fried at both temperatures, and the selected times for 120 °C were 30 and 60 min and the times for 170 °C were 15 and 30 min, time being a qualitative factor. A full-factorial design was performed (2^2^) with three replicates per point to assess the effect of the temperature and time of cooking and the possible interaction between these two factors. The levels and the processing conditions are shown in Table 1.

The domestic sautéing was performed at the Food Torribera Campus, University of Barcelona (Santa Coloma de Gramenet, Spain). The pan was heated on an electrical cooking plate (180 mm diameter, 1500 W, model Encimera EM/30 2P, Teka^®^, Madrid, Spain) until the required temperature was reached. The temperature was monitored with a laser thermometer (error: ±1 °C, ScanTemp 410, TFA Dostmann GmbH & Co. KG, Wertheim, Germany) and maintained by turning the heat up or down as necessary. When the target temperature was achieved, 20 g of EVOO were added to the pan and heated for the chosen time. The pan was then removed from the heat and after a short cooling period, the oil was stored in a vacuum bag at −20 °C until extraction. The oxygen or light were not controlled to mimic the process carried out in a normal kitchen.

### 2.4. Polyphenol Extraction and Analysis

#### 2.4.1. Polyphenol Extraction

The liquid-liquid extraction of phenolic compounds was performed following the method proposed by Kalogeropoulos et al. (2007) with minor modifications [26]. All of the extraction process was carried out over an ice bed. Briefly, 0.5 g of EVOO was suspended with 5 mL of methanol in a 10 mL centrifuge tube and stirred for 30 s. It was centrifuged for 3 min at 3000 rpm and 4 °C. The methanolic fraction was then transferred into a flask and the extraction was repeated. Both methanolic fractions were combined and evaporated under a reduced pressure. The residue was reconstituted with 2 mL of acetonitrile and washed twice with 2 mL of hexane. The acetonitrile was evaporated under a reduced pressure and the residue was reconstituted with 800 μL of MeOH:H_2_O (4:1 *v*/*v*), filtered with Polytetrafluoroethylene syringe filters (0.2 µm), and was transferred to an amber glass vial and stored at −80 °C until analysis.

#### 2.4.2. Polyphenol Analysis by UPLC-ESI-QqQ-MS/MS

The identification and quantification of phenolic compounds, except oleocanthal, oleacein and oleuropein and ligstroside aglycones, was performed following the method proposed by Suárez et al. (2008) with minor modifications [27], using an AcquityTM UPLC (Waters; Milford, MA, USA) coupled to an API 3000 triple-quadruple mass spectrometer (PE Sciex, Framingham, MA, USA) with a turbo ion spray source. The separation of compounds was achieved using an Acquity UPLC^®^ BEH C18 Column (2.1 × 50 mm, i.d., 1.7 µm particle size) (Waters Corporation^®^, Wexford, Ireland) and an Acquity UPLC^®^ BEH C18 Pre-Column (2.1 × 5 mm, i.d., 1.7 µm particle size) (Waters Corporation^®^, Wexford, Ireland). The exact chromatographic conditions were as detailed elsewhere [28].

The quantification of oleocanthal, oleacein, oleuropein aglycone, and ligstroside aglycone was performed using a methodology proposed by Sánchez de Medina et al. (2017) with some modifications [29]. Separation was achieved using an Acquity UPLC^®^ BEH C18 Column (2.1 × 50 mm, i.d., 1.7 µm particle size) (Waters Corporation^®^, Wexford, Ireland) and Acquity UPLC^®^ BEH C18 Pre-Column (2.1 × 5 mm, i.d., 1.7 µm particle size) (Waters Corporation^®^, Wexford, Ireland). The exact chromatographic conditions were as detailed elsewhere [28].

Ionization was performed using an electrospray (ESI) interface operating in the negative mode [M − H], and all of the compounds were monitored in the multiple reaction monitoring mode (MRM). The exact ionization and spectrometric conditions are detailed in the previous study [28], and the energies and retention times for each analyzed compound are shown in Tables S1 and S2. The system was controlled by Analyst version 1.4.2 software supplied by Applied Biosystems (Waltham, MA, USA). 

Quantification was performed by an external standard calibration method, standards showed linearity in the concentration range 1–20 mg/L. Quantification was performed using oleuropein for hydroxydecarboxymethyl oleuropein aglycone (HDCM-OA), hydroxyoleuropein aglycone (HOA), elenolic acid, and hydroxyelenolic acid; hydroxytyrosol for hydroxytyrosol and hydroxytirsol acetate; the respective standards for ferulic acid, *p*-coumaric acid, pinoresinol, apigenin and luteolin were used; and oleocanthal was used for oleocanthal, ligstroside aglycone, oleacein, and oleuropein aglycone. 

### 2.5. Statistical Analysis

The statistical differences between samples of EVOO taken in different cooking conditions were analyzed by Statistica version 10.0.228.8 (StatSoft Inc., Tulsa, OK, USA) using the factorial ANOVA test. The assumption of normalization was graphically checked. To assess the importance of the contributing factors, multiple linear regressions were calculated. The form of the regression is as follows:

(1) Concentration = β_0_ + β_1_·T + β_2_·t + β_3_·Tt

where T stands for temperature, t stands for time, and each β is the contribution of these factors. If its *p*-value is lower than 0.05, then β is significantly different from 0. The statistic *R*^2^ is adjusted to the size of the model and can decrease if insignificant factors are added [30]. This parameter measures the proportion of the total variability explained by the model [31,32]. Even if the factors were not statistically significant, they were added to the model as confusing variables, and it was assessed if the model was more accurate with or without them. The model with the largest adjusted *R*^2^ was selected. In order to build the model, the low temperature was the point −1 and the high temperature point was +1, and the same was applied for the short (−1) and long (+1) cooking time. Then, the value of β multiplied per 2 is the difference between the two levels of a factor.

## 3. Results and Discussion

### 3.1. Total Polyphenols

The concentrations of different polyphenols and of the groups found in raw and cooked EVOO samples are presented in Table 2. 

When EVOO was heated in a pan, the sumatory of polyphenolic content decreased by around 40% at the low temperature (120 °C) and 75% at the high temperature (170 °C). Casal et al. (2010) reported a decrease of 50% in the total phenolic content, measured by the Folin-Ciocalteu method, after heating olive oil in a domestic deep-fat fryer at 170 °C for 3 h. [23]. However, in this study, the oil was deep fried so the samples were less exposed to oxygen and light, which may explain why the results are different to those presented here. Moreover, Folin-Ciocalteau methods are not selective, so this variation is not measuring only the phenol content, but also other reducing compounds. For this reason, it is also difficult to compare the results with those showed by one recent study, in which the degradation of the total phenolic content during a sautéing process was evaluated. The authors showed a decrease of approximately 50% of the antioxidant capacity measured by the Folin-Ciocalteau method after sautéing typical Mediterranean vegetables (potato, eggplant, tomato, and pumkin) for 10 min at 100 °C [33].

**Figure 1:**
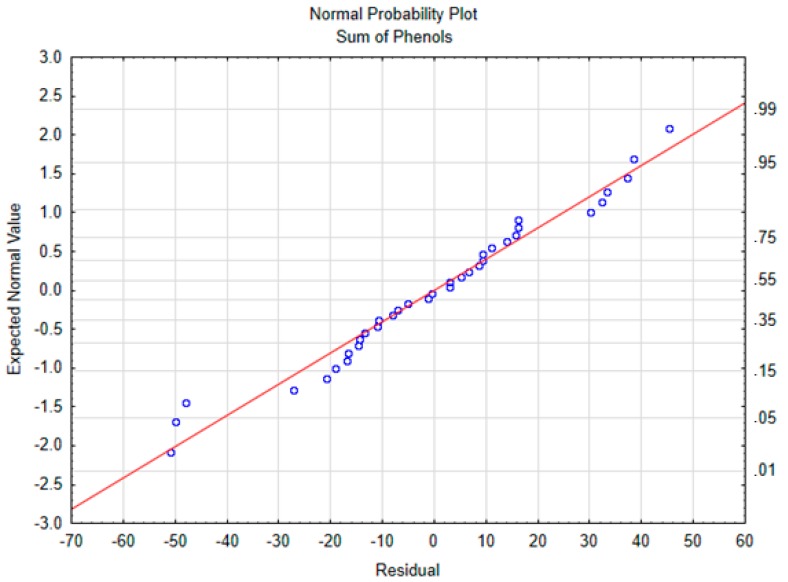
Normal probability plot of the sum of phenols.

For the ANOVA and multiple regression models, the normality of residuals was verified. To check this assumption, normal probability plots of the residuals were plotted for each compound. The graph for the sum of polyphenols is shown in Figure 1. The results of the ANOVA test and the linear regression models are shown in Table 3. The temperature was mainly responsible for the polyphenols depletion and there were no significant effects from time or the interaction. These results are in accordance with those reported by Goulas et al. (2015), who showed that heating the oil at 180 °C for 1 h or for 5 h made no difference in polyphenol content decrease [8].

The model for the sum of polyphenols was great fitted, with a *R*^2^ of 0.97, in which 97% of the variance is explained by the model. The slope for temperature was significantly different from 0, suggesting that a longer cooking period does not change the polyphenolic fraction when the EVOO is processed only once. As the low level is −1 and the high level is +1, and the β of the temperature is −131, then cooking using a high temperature decreased the polyphenol content 232 mg/kg more than applying a moderate temperature, which represents 27% of the raw EVOO concentration.

### 3.2. Secoiridoids

Secoiridoids are the largest group of EVOO polyphenols. Secoiridoids include oleuropein, ligstrosides, and their derivatives. Some of them have been reported to have important benefits to health, such as oleocanthal or oleacein [34]. Oleocanthal has demonstrated anti-inflammatory effects [35] and a protective role against some diseases, such as Alzheimer disease [34], and oleacein has proven to protect against cardiovascular diseases, reducing hypertension [36] and inhibiting neutrophils adhesion [37].

During the cooking process, secoiridoids decreased 45% at the low temperature and 70% at the high temperature. Among this group, a different behavior was observed in hydroxyelenolic acid, which is not a polyphenol but a related compound produced by the ester breakdown of ligstroside, oleuropein, and their aglycones [38]. Thus, the formation of hydroxyelenolic acid was enhanced by processing at a moderate temperature. However, a longer cooking period and a higher temperature promoted its degradation, giving a lower concentration.

According to ANOVA analysis, the factor responsible for the depletion of secoiridoids was temperature. However, different results were found for each secoiridoid, in which oleacein and oleuropein aglycone were also affected by the interaction of time and temperature, and hydroxydecarboxymethyl oleuropein aglycone and hydroxyoleuropein aglycon were affected by all of the evaluated factors. These results are in accordance with those reported by Attya et al. (2010), in which heating at 90 °C was shown to cause almost no degradation of oleocanthal and oleacein in EVOO, but at 170 °C the concentration of both compounds was reduced by half, reflecting the major role played by temperature in polyphenol degradation [16].

The models were properly fitted for most of the secoiridoids analyzed, however, oleocanthal showed a *R*^2^ of 0.7 because of its high reactivity, which also prompted the development of a new method for its specific analysis [29]. Oleocanthal presents keto-enolic tautomerism, which impeded its proper analysis, as it reacts with the solvent during the chromatographic separation. Oleocanthal may have reacted more in some samples than others, but even with only a 70% model fit, the result indicates that the cooking time did not change the oleocanthal concentration. In contrast, it decreased by 100 mg/kg of oil after cooking at a high temperature compared to the moderate temperature.

In the case of the sum of secoiridoids, the ANOVA results showed that the interaction factor was not significant, although in the multiple regression model the β for the interaction was different to 0, indicating that there was an effect. This difference occurred because the test used for ANOVA and the test used for the regression model were different: ANOVA applies a *F*-test, and, for the regression models, a t-test is used. Despite the ANOVA result giving a *p*-value of over 0.05, it showed a trend that this factor had an effect on secoiridoid degradation (*p*-value = 0.052).

According to the slopes of the models, the temperature was mainly responsible for the depletion of seicoiridoids during a domestic sautéing process. Slopes were analyzed as a percentage of the initial concentration (in raw EVOO), as the initial concentration for each polyphenol differed substantially, making it difficult to compare the models between the different polyphenols. The results are shown in Table 3. The slopes represent the values ranging from 12% (ligstroside aglycone) to 20% (oleuropein aglycone) of their original concentrations. The most different one was oleocanthal, which showed just a 6.5% depletion and withstands better the temperature than oleuropein aglycone. The compounds with a *o*-diphenol group were the most reactive, with a slope representing between 18% (hydroxydecarboxymethyl oleuropein aglycone) an 20% (oleacein) of their initial concentration. On the other hand, oleocanthal and ligstroside aglycone (compounds with just one hydroxyl group) showed less reactivity. *ortho*-Diphenols are the most reactive, as they can be converted easily to *ortho*-quinones through a radical reaction [39,40,41]. Also, the intermediates of the reaction are radicals too, so they stabilized by the hydroxyl in the ortho position [40]. This rapid conversion may be responsible for the higher degradation compared to single phenols. This difference in the reactivity is also reflected in the activation energy, in which oleocanthal presents lower than oleacein because a higher temperature change is needed to degrade oleocanthal at the same rate as oleacein [16].

As mentioned above, hydroxyelenolic acid was an exception, its concentration was affected by the cooking time and the interaction between time and temperature, but not the temperature alone. For this compound and for elenolic acid, the time factor had a positive effect as the slopes were positive, indicating that frying with EVOO for longer periods may increase their concentration. Although the hydroxyelenolic acid model was not well fitted, the elenolic acid model showed an 82% fitness result. Like hydroxyelenolic acid, elenolic acid is not a phenol, but a derivative of oleuropein and ligstroside aglycones. Thus, despite a long cooking process degrading some of the polyphenols, it can enhance some related and new compounds.

### 3.3. Phenolic Alcohols and Others

Phenolic alcohols, mainly hydroxytyrosol and hydroxytyrosol acetate, are derivates from oleuropein, like the secoiridoids, but as their chemical behaviors are different, they are classified in a different group [27,28].

**Figure 2:**
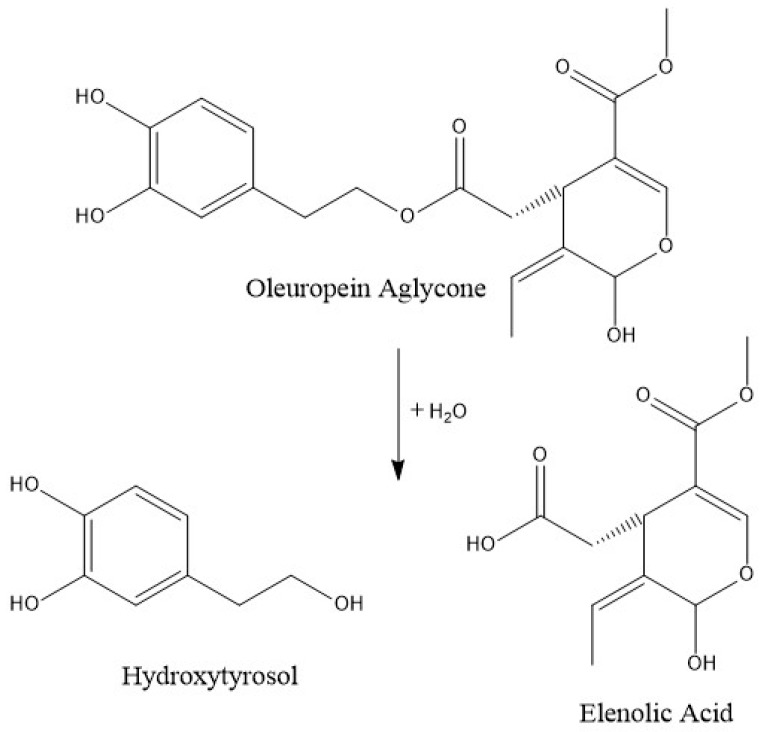
Normal probability plot of the sum of polyphenols.

When frying at a low temperature for a short time, only 9% of phenolic alcohols decreased, although a depletion of 85% (90% in the case of hydroxytyrosol), when applying a high temperature and a long cooking time, was observed. At a low or moderate temperature, the hydroxytyrosol degradation, formed by the ester breakdown of oleuropein and its aglycones (Figure 2), may be counteracted by the rate of its generation. However, at a high temperature, its degradation is more likely to occur, resulting in a substantial reduction. Similar results are observed by Ramírez-Anaya et al. (2019), who showed that after sautéing typical Mediterranean vegetables at 100 °C, the hydroxytyrosol content only decreased between 25% and 50% [33]. Furthermore, a similar behavior was found by Krichene et al. (2015), who showed an increase in hytroxytyrosol concentration during the first months of storage due to the transformation of oleuropein and derivates in the compound, but after some months there was a high decrease in its concentration [42].

The concentration of phenolic alcohols was affected by temperature and time and hydroxytyrosol was also affected by their interaction, however, the β value for the temperature factor was higher, indicating that it is mainly responsible for their degradation.

Hydroxytyrosol was the most degraded compound by temperature, its slope represents 30% of its initial concentration. Thus, the amount of hydroxytyrosol diminished greatly—about 60% of the hydroxytyrosol concentration in the raw EVOO cooked at a high temperature compared to the EVOO cooked at a low temperature. So, cooking at low temperature should be recommended due to the fact that, according to the European Food and Safe Authority, it protects low-density lipoproteins (LDL) from oxidative damage [43] that have proven health effects.

The minor groups of polyphenols in EVOO are phenolic acids, lignans, and flavones. There was no possibility to build a model for those groups because of their low concentration. The only model properly fitted (>80%) for flavones was luteolin that was mainly affected by temperature. In the case of lignans, the only compound present in quantifiable amounts was pinoresinol, which increased during cooking probably because of the transformation of 1-acetoxypinoresinol and because of its high temperature stability [44].

## 4. Conclusions

In this work, we determined changes in the EVOO polyphenolic profile during a domestic sautéing process commonly used in the Mediterranean diet, simulating the cooking conditions of a home kitchen, without the control of light or oxygen. The cooking temperature was the most important factor in the degradation of EVOO polyphenols. In the case of time, it was only a significant factor for some polyphenolic compounds and was not a significant factor for the sum of the polyphenols. Sautéing at a low temperature changes the polyphenolic profile of EVOO by increasing the concentration of hydroxyelenolic acid and depleting other compounds. Besides, this oil would still have the amount of polyphenols, with values higher than 250 mg/kg of hydroxytyrosol, tyrosol, or the derivates necessary to inhibit LDL oxidation [43]. Furthermore, research is needed to determine if there are differences in EVOO polyphenol degradation when proteins or complex sugars are present and whether the presence of these phenolics for their antioxidant properties could avoid the formation of secondary undesirable compounds that originated from the cooking and food processing.

## Figures and Tables

**Table 1:** Levels and conditions of the full factorial design.

Experiment	Temperature (Level)	Time (Level)	Temperature	Time
1	–1	–1	120 °C	30
2	1	–1	170 °C	15
3	–1	1	120 °C	60
4	1	1	170 °C	30

**Table 2:** Polyphenolic concentration of raw and processed extra-virgin olive oil (EVOO) expressed in mg/kg of EVOO.

Group/Compound	Raw	↓T ↓t	↓T ↑t	↑T ↓t	↑T ↑t
**Sum of phenols**	860 ± 22	487 ± 29	498 ± 32	240 ± 19	218 ± 12
**Secoiridoids**	835 ± 22	466 ± 30	481 ± 31	231 ± 20	213 ± 12
Ligstroside aglycone	368 ± 7	190 ± 13	193 ± 11	94 ± 21	97 ± 7
Oleocanthal	81 ± 4	51 ± 3	53 ± 5	41 ± 3	41 ± 3
Oleuropein aglycone	79 ± 2	45 ± 3	47 ± 3	15 ± 2	12 ± 1
Oleacein	252 ± 9	134 ± 13	139 ± 15	46 ± 6	32 ± 4
HDCM-OA	23.6 ± 0.9	21 ± 2	22 ± 3	16 ± 2	9 ± 1
HOA	3.2 ± 0.2	2.3 ± 0.3	3.3 ± 0.5	2.3 ± 0.3	0.7 ± 0.1
Elenolic acid	25.1 ± 0.2	16 ± 2	16 ± 1	10 ± 1	10.9 ± 0.6
Hydroxyelenolic acid	1.9 ± 0.1	8 ± 1	8 ± 1	6.7 ± 0.8	9.1 ± 0.9
**Phenolic alcohols**	19.6 ± 0.5	18 ± 1	14 ± 1	5.9 ± 0.6	2.8 ± 0.2
Hydroxytyrosol acetate	4.5 ± 0.2	3.9 ± 0.3	4.0 ± 0.2	1.8 ± 0.2	1.4 ± 0.1
Hydroxytyrosol	15.2 ± 0.7	14 ± 1	10.0 ± 0.9	4.1 ± 0.5	1.5 ± 0.2
**Flavonoids**	1.8 ± 0.2	1.3 ± 0.4	1.3 ± 0.4	0.79 ± 0.08	0.86 ± 0.05
Apigenin	0.61 ± 0.04	0.7 ± 0.4	0.7 ± 0.4	0.48 ± 0.05	0.54 ± 0.03
Luteolin	1.16 ± 0.15	0.54 ± 0.08	0.61 ± 0.06	0.31 ± 0.04	0.32 ± 0.02
**Phenolic acids**	3.7 ± 0.3	1.4 ± 0.7	1.2 ± 0.4	1.2 ± 0.3	0.8 ± 0.2
Ferulic acid	3.3 ± 0.3	0.9 ± 0.7	0.7 ± 0.4	0.7 ± 0.3	0.4 ± 0.2
*p*-Coumaric acid	0.45 ± 0.01	0.49 ± 0.06	0.47 ± 0.04	0.47 ± 0.04	0.40 ± 0.02
**Lignans**	0.44 ± 0.05	0.48 ± 0.07	0.49 ± 0.05	0.60 ± 0.06	0.64 ± 0.07
Pinoresinol	0.44 ± 0.05	0.48 ± 0.07	0.49 ± 0.05	0.60 ± 0.06	0.64 ± 0.07

HDCM-OA: Hydroxydecarboxymethyloleuropein Aglycone; HOA: Hydroxyoleuropein Aglycone; T: temperature; t: time; ↑ high level of the factor; ↓ low level of the factor.

**Table 3:** Statistical results of the ANOVA and the lineal models.

Group/Compound	*R* ^2^	β_0_	Temperature		Time		Interaction	
			*F*-Value	β_1_	*F*-Value	β_2_	*F*-Value	β_3_
**Sum of phenols**	0.968	360.8 *	1038.8 *	−131.9 *	0.458	-	3.87	−8.05
**Secoiridoids**	0.965	347.9 *	946.9 *	−125.8 *	0.057	-	4.04	−8.22 *
Ligstroside aglycone	0.927	143.2 *	427.4 *	−47.8 *	0.443	-	0.0018	-
Oleocanthal	0.703	46.5 *	81.0 *	−5.31 *	0.603	-	0.232	-
Oleuropein aglycone	0.973	29.7 *	1276.1 *	−16.2 *	0.402	−0.287	8.96 *	−1.35 *
Oleacein	0.955	87.9 *	736.9 *	−48.6 *	1.59	−2.26	6.83 *	−4.68 *
HDCM-OA	0.845	17.2 *	144.3 *	−4.34 *	15.5 *	−1.42 *	33.7 *	−2.10 *
HOA	0.888	2.18 *	369.9 *	−0.660 *	0.718	−0.147 *	142.7 *	−0.645 *
Elenolic acid	0.824	13.2 *	159.8 *	−2.99 *	4.26 *	0.488 *	0.678	-
Hydroxyelenolic acid	0.444	7.92 *	0.002	-	17.1 *	0.654 *	11.9 *	0.546 *
**Phenolic alcohols**	0.978	10.1 *	1427.7 *	−5.78 *	124.6 *	−1.71 *	1.36	0.178
Hydroxytyrosol acetate	0.964	2.77 *	916.5 *	−1.18 *	3.28	−0.0705	12.6 *	−0.138 *
Hydroxytyrosol	0.971	7.37 *	1026.5 *	−4.60 *	129.9 *	−1.64 *	4.86 *	0.316 *
**Flavonoids**	0.396	1.06 *	23.1 *	−0.236 *	0.723	-	0.0038	-
Apigenin	0.121	0.615 *	5.54 *	−0.105 *	0.265	-	0.0648	-
Luteolin	0.855	0.446 *	202.4 *	−0.131 *	4.17 *	0.0187 *	2.44	−0.0144
**Phenolic acids**	0.090	1.15 *	3.35	−0.147	3.08	−0.141	0.0288	−0.0136
Ferulic acid	0.085	0.682 *	3.27	−0.133	2.97	−0.127	0.0158	−0.00927
*p*-Coumaric acid	0.111	0.463 *	3.43	−0.013	3.60	−0.0138	0.354	−0.00433
**Lignans**	0.520	0.553 *	39.2 *	0.068 *	1.16	0.0116	0.583	0.00827
Pinoresinol	0.526	0.553 *	39.2 *	0.068 *	1.16	0.0116	0.583	-

* Statistically significant difference (*p*-value < 0.05) for ANOVA (*F*-value) and for the linear regression analyses (β).

## Supplementary Materials

The following are available online at https://www.mdpi.com/2076-3921/9/1/77/s1, Table S1: Ionization conditions for compounds analyzed with the method 1, Table S2: Ionization conditions for compounds analyzed with the method 2.
