# Supplementary material for: Domestic Sautéing with EVOO: Change in the Phenolic Profile
Source: Antioxidants (Basel). 2020 Jan 16;9(1):77. doi: 10.3390/antiox9010077 (PMC7022658; doi:10.3390/antiox9010077)
Supplement: Supplementary file 1 [file antioxidants-09-00077-s001.pdf]

Table S1. Ionization conditions for compounds analyzed with the method 1.

Compound	Transitionn	Declustering Potential	Focusing Potential	Entrance Potential	Collision Energy	Dwell Time (ms)	Retention Time (min)
HDCM-OA	335/199	–30	–140	–10	–30	30	7.4
HOA	393/257	–30	–140	–10	–30	20	8.1
Elenolic acid	241/127	–30	–140	–10	–30	50	6.3
Hydroxyelenolic acid	257/137	–30	–140	–10	–30	30	5.9
Hydroxytyrosol acetate	195/153	–30	–140	–10	–30	20	10
Hydroxytyrosol	153/123	–40	–250	–10	–20	25	1.1
Apigenin	269/117	–70	–200	–10	–50	30	9.2
Luteolin	285/133	–100	–340	–10	–50	30	8.3
Ferulic acid	193/134	–40	–170	–10	–20	20	5.2
p-Coumaric acid	163/119	–40	–150	–10	–25	30	4.3
Pinoresinol	357/151	–60	–180	–8	–25	20	8.7

Table S2: Ionization conditions for compounds analyzed with the method 2

Compound	Transitionn	Declustering Potential	Focusing Potential	Entrance Potential	Collision Energy	Dwell Time (ms)	Retention Time (min)
Ligstroside aglycone	361/291	–40	–170	–5	–10	150	2.7
Oleocanthal	303/285	–40	–170	–5	–10	50	2.4
Oleuropein aglycone	377/275	–45	–140	–5	–15	150	2.5
Oleacein	319/69	–30	–170	–5	–30	50	2.3
